# Challenges, opportunities, and future perspectives of portable field endoscopy

**DOI:** 10.1186/s40779-025-00666-4

**Published:** 2025-11-17

**Authors:** Ke He, Shu-Yi Wang, Jun Ren

**Affiliations:** 1 Minimally Invasive Tumor Therapies Center, the Affiliated Guangdong Second Provincial General Hospital of Jinan University, Guangzhou, 510317 China; 2 Department of Emergency, Shanghai Tenth People’s Hospital, School of Medicine, Tongji University, Shanghai, 200072 China; 3 National Clinical Research Center for Interventional Medicine, Shanghai, 200032 China; 4 Department of Cardiology, Shanghai Institute of Cardiovascular Diseases, Zhongshan Hospital Fudan University, Shanghai, 200032 China

**Keywords:** Portable endoscopy, YunSendo system, Single-use devices, Battlefield medicine, Infection control, Gastrointestinal endoscopy

Traditional in-hospital endoscopy systems have long served as the cornerstone of gastrointestinal diagnostics and therapy, offering high-resolution imaging, versatile therapeutic capacity, and seamless integration into established hospital workflows. These advantages, however, are closely tied to the infrastructure they depend on. Conventional endoscopy towers are bulky and require reliable power, water supply, and sterilization facilities, resources rarely available in battlefields, disaster zones, or remote clinics. Beyond portability concerns, infection control represents another critical challenge. Reusable endoscopes demand strict high-level disinfection with specialized equipment and trained personnel. Yet even in high-resource hospitals, lapses in reprocessing have caused outbreaks of duodenoscope-associated infections, including multidrug-resistant organisms [1]. In addition, the financial and logistical burdens are substantial: maintenance, consumables, and reprocessing often account for costs comparable to or exceeding those of emerging single-use platforms [2]. Moreover, pandemic conditions magnify these vulnerabilities, as endoscopic procedures generate aerosols and expose staff to respiratory and gastrointestinal secretions, intensifying the risk of pathogen transmission [3]. Collectively, these limitations expose a fundamental trade-off, while traditional hospital-based systems remain indispensable for comprehensive care, their drawbacks make them poorly suited for austere, outbreak-prone, or resource-limited environments. This recognition has catalyzed worldwide efforts to develop portable, single-use, and disposable endoscopy platforms emphasizing mobility, sterility, and adaptability.

To address these unmet needs, Sun et al. [4] developed the YunSendo portable field endoscope was published in *Military Medical Research*. Weighing only 13.8 kg, YunSendo demonstrated performance comparable to the Olympus system across key metrics, confirming its suitability for urgent scenarios. This endoscopic device integrates portability with single-use disposability to overcome the infection-control challenges inherent to reusable systems. By eliminating the need for reprocessing and thereby minimizing cross-infection risks, an advantage especially critical during outbreaks and for immunocompromised patients, the YunSendo system aligns with the post-COVID-19 global shift toward biosafety-driven, single-use endoscopy solutions. Early clinical testing demonstrated non-inferior image quality and lesion detection rates compared to conventional Olympus towers, with only modest increases in procedure time [4]. These results highlight YunSendo’s potential as a diagnostic and limited therapeutic tool in environments where conventional towers are impractical. Its lightweight and modular structure make it especially suited to combat operations, shipboard missions, and disaster relief, where gastrointestinal disease remains a major cause of non-combat morbidity. Another exciting advantage lies in its tele-endoscopy capability. Lightweight hardware combined with digital connectivity allows frontline medics in remote areas to perform diagnostic procedures while receiving real-time specialist guidance via telecommunication. When coupled with AI-assisted image interpretation, this could bridge expertise gaps and facilitate timely interventions in resource-limited or geographically isolated settings.

YunSendo is already compatible with gastrointestinal and bronchoscopic scopes and can potentially expand to cystoscopy, laparoscopy, or thoracoscopy. This modular adaptability could transform it into a universal portable endoscopy hub, reducing the need for multiple bulky platforms, a crucial benefit in space- and weight-constrained field hospitals. Its disposable design also carries important educational value. Students and trainees can practice procedures without risking damage to those costly reusable conventional systems or exposing patients to infection. This feature democratizes endoscopy training in low-resource environments and contributes to strengthening global gastrointestinal care capacity.

From a broader endoscopic perspective, the development of YunSendo parallels a global paradigm shift toward portable and infection-resilient device technologies. Over the past decade, increasing awareness of cross-contamination risks and infrastructure constraints has accelerated innovation in lightweight, self-contained, or disposable systems. Internationally, several analogous systems have defined this evolving landscape. The EXALT Model D duodenoscope (Boston Scientific) pioneered single-use therapeutic endoscopy for endoscopic retrograde cholangiopancreatography (ERCP), achieving performance parity with reusable scopes while eradicating reprocessing-related infection risk. The Ambu aScope series, now widely adopted in intensive care and anesthesia settings, emphasizes sterility, immediate readiness, and simplified operator training [5, 6]. In parallel, portable gastrointestinal “suitcase” systems from Japan, South Korea, and Germany integrate imaging, light source, and power supply into compact mobile units. These enable on-site diagnostics but remain limited by size, cost, and dependence on reusable components [7]. At the lower-cost end, smartphone-based endoscopy platforms such as those reported by Bae et al. [8] offered remarkable mobility for triage and education, though image resolution and therapeutic scope remain modest. Capsule-based systems extend remote diagnostic reach and exemplify the convergence of portability, disposability, and digital monitoring in endoscopic care.

Within this global framework, YunSendo may represent a strategically positioned intermediate platform, offering superior structural robustness and reduced weight compared with conventional portable suitcase endoscopes, while exhibiting greater clinical functionality than smartphone-assisted or capsule-based modalities. Its 13.8 kg modular framework seeks to balance mobility, biosafety, and performance in field and emergency scenarios. Nevertheless, several limitations persist. YunSendo currently trails leading systems in therapeutic breadth, anti-fogging, and control precision [4]. Validation has so far focused on experienced operators (> 10,000 cases), leaving questions about usability among general clinicians. Standardized training modules and simulation-based practice will therefore be essential. Moreover, while effective for biopsy, polypectomy, and hemostasis, it has yet to be validated for complex interventions such as stenting or large-volume bleeding control. The open-label, self-controlled trial design and testing under hospital conditions also limit extrapolation to genuine field environments.

Beyond performance, the single-use model raises sustainability and logistical challenges. Manufacturing scalability, supply-chain resilience, and waste management must be optimized, particularly for military or disaster contexts where disposal infrastructure is limited. Economic viability will depend on infection risk, case volume, and existing reprocessing infrastructure, necessitating rigorous multicenter cost-effectiveness and lifecycle analyses.

Moving forward, next-generation YunSendo development should prioritize technical refinements, enhanced anti-fogging, improved ergonomics, broader therapeutic compatibility, and AI-assisted visualization, alongside rigorous multicenter validation. Testing in extreme environments (altitude, desert, maritime, cold) should be required for military adoption, while sustainable design using biodegradable or recyclable materials will address ecological and supply-chain challenges. Future iterations may incorporate real-time tele-endoscopy and 5G-enabled remote mentoring, transforming field care through cloud-based diagnostics and AI quality control. Considering its modular architecture, YunSendo may be developed into a multifunctional endoscopy hub encompassing gastrointestinal, respiratory, urological, gynecological, and many minor surgical applications. Integration with portable energy modules, wireless data encryption, and standardized operator training could further expand its readiness for battlefield and disaster medicine. Collectively, these innovations will position YunSendo as a versatile, intelligent, and sustainable platform shaping the next frontier of civilian and military field healthcare.

## Data Availability

All relevant data and materials used for the generation of this work will be made available upon request to the corresponding author.

## Abbreviation

ERCP: Endoscopic retrograde cholangiopancreatography

## References

[1] Muthusamy VR, Bruno MJ, Kozarek RA, Petersen BT, Pleskow DK, Sejpal DV, et al. Clinical evaluation of a single-use duodenoscope for endoscopic retrograde cholangiopancreatography. Clin Gastroenterol Hepatol. 2020;18(9):2108-17.e3. 31706060 10.1016/j.cgh.2019.10.052

[2] Deasy KF, Sweeney AM, Danish H, O’reilly E, Ibrahim H, Kennedy MP. Single use or disposable flexible bronchoscopes: bench top and preclinical comparison of currently available devices. J Intensive Care Med. 2023;38(6):519–28. 36609193 10.1177/08850666221148645 PMC10114257

[3] Chiu PWY, Ng SC, Inoue H, Reddy DN, Ling Hu E, Cho JY, et al. Practice of endoscopy during COVID-19 pandemic: position statements of the Asian Pacific Society for Digestive Endoscopy (APSDE-COVID statements). Gut. 2020;69(6):991–6. 32241897 10.1136/gutjnl-2020-321185 PMC7211066

[4] Sun G, Zeng JQ, Wu JL, Wang SF, Peng LH, Yan B, et al. Evaluation and application of an innovative portable field endoscope: addressing battlefield and biosafety concerns. Mil Med Res. 2025;12(1):57. 40954480 10.1186/s40779-025-00644-w PMC12434913

[5] Ehrlich D, Muthusamy VR. Device profile of the EXALT model D single-use duodenoscope for endoscopic retrograde cholangiopancreatography: overview of its safety and efficacy. Expert Rev Med Devices. 2021;18(5):421–7. 33855920 10.1080/17434440.2021.1917990

[6] Boyd S, Murphy CJ, Snyman L. Single-use vs. reusable flexible bronchoscopes for airway management and in critical care: a narrative review. Anaesthesia. 2025;80(2):197–204. 39344667 10.1111/anae.16430 PMC11726266

[7] Zou WB, Zhang T, He C, Duan XD, Liu C, Li ZS, et al. A novel portable upper gastrointestinal endoscopy system with complete functions of both diagnosis and treatment. Endoscopy. 2023;55(S1):E9-10. 36084941 10.1055/a-1919-4443 PMC9812668

[8] Bae JK, Vavilin A, You JS, Kim H, Ryu SY, Jang JH, et al. Smartphone-based endoscope system for advanced point-of-care diagnostics: feasibility study. JMIR Mhealth Uhealth. 2017;5(7):e99. 28751302 10.2196/mhealth.7232 PMC5553006

